# Purulent Pericarditis in End-Stage Renal Disease: A Rare Case of Citrobacter freundii Infection

**DOI:** 10.7759/cureus.62308

**Published:** 2024-06-13

**Authors:** Hugo Goncalves, Rosa Sá, Francisco de Oliveira Simões, Rui M Domingues, Narciso Oliveira, Teresa Pimentel

**Affiliations:** 1 Division of Rheumatology, Unidade Local de Saúde de Braga (ULS de Braga), Braga, PRT; 2 Division of Oncology, Unidade Local de Saúde de Braga (ULS de Braga), Braga, PRT; 3 Division of Internal Medicine, Unidade Local de Saúde de Braga (ULS de Braga), Braga, PRT; 4 Internal Medicine, Hospital de Braga, Braga, PRT

**Keywords:** immunosuppression, end-stage renal disease, citrobacter freundii, tamponade, purulent pericarditis

## Abstract

Background: Purulent pericarditis is a rare but life-threatening condition, particularly challenging when it occurs in immunocompromised individuals.

Case report: We present the case of a 68-year-old man with end-stage renal disease who developed purulent pericarditis secondary to *Citrobacter freundii* infection. Despite initial challenges in diagnosis and management, the patient showed a favorable response to antibiotic therapy.

Conclusions: This case highlights the importance of prompt recognition and treatment of purulent pericarditis, especially in patients with underlying immunosuppression and comorbidities.

## Introduction

Acute pericarditis is the most prevalent inflammatory heart disease, being more common than acute myocarditis and acute endocarditis [1] and being responsible for 5% of non-ischemic chest pain in emergency departments [2]. Although generally manifesting with a benign and self-limited course, there are cases where short-term complications arise, with the most serious and potentially fatal being cardiac tamponade due to rapid expansion of the pericardial effusion compromising cardiac chamber filling and impairing adequate cardiac output [1,3].

In developed countries, 80-90% of acute pericarditis cases are idiopathic, most likely of viral etiology [2]. The remaining cases are divided among pericarditis associated with connective tissue diseases, post-myocardial infarction pericarditis, and neoplastic pericarditis [2]. Purulent pericarditis, once much more prevalent before the advent of antibiotic therapy, has become increasingly rare [4]. Among purulent pericarditis cases, the most frequently isolated bacteria are Gram-positive *cocci*, specifically *Staphylococcus spp.* and *Streptococcus spp.* [4]. Although rare, purulent pericarditis is a severe form of pericarditis characterized by the presence of pus in the pericardial sac. It usually results from bacterial infections and can rapidly progress to life-threatening conditions if not promptly treated.

We present a case of a 68-year-old man with known chronic pericardial effusion experiencing exacerbation of symptoms and developing obstructive shock due to cardiac tamponade.

## Case presentation

A 68-year-old man with end-stage renal disease (ESRD) had multiple emergency department (ED) visits for chest pain for the past two months. Acute coronary syndrome was always excluded, and the pain was interpreted as secondary to musculoskeletal pathology. The patient's medical history is significant for hypertension and autosomal dominant polycystic kidney disease (ADPKD), and he has a relevant family history, with his father also affected by the last condition. The patient's usual medications included antihypertensives (Nifedipine and losartam), phosphate binders (sevelamer), erythropoiesis-stimulating agents (darbopoietin), and vitamin D supplementation (calcitriol).

The patient returned to the ED due to a new episode of precordial chest pain with pleuritic characteristics. Additionally, he reported an isolated fever episode accompanied by anorexia and generalized myalgias. During evaluation in the emergency department, he experienced a syncopal episode. On physical examination, the patient was hypotensive, with a blood pressure of 69/53 mmHg, a heart rate of 112/min, a temperature of 37.2ºC, and a respiratory rate of 15/min. The patient also had muffled cardiac sounds and lower limb edema, and, upon evaluation by cardiology with point-of-care ultrasound (POCUS), a large-volume pericardial effusion was noted.

Analytically, there was an elevation of liver enzymes consistent with ischemic hepatitis (aspartate aminotransferase of 9716 U/L [laboratory reference values 12-40 U/L]; alanine aminotransferase of 4620 U/L [laboratory reference values 7-40 U/L]) and a slight elevation of myocardial necrosis markers (Troponin I of 0.057 ng/mL [laboratory reference values <0.045 ng/mL]). Additionally, there was an elevation of inflammatory parameters, namely reactive C protein, of 137.70 mg/L. The detailed laboratory findings are presented in Table 1.

**Table 1:** Laboratory values at admission AST: aspartate aminotransferase, ALT: alanine aminotransferase, ALP: alkaline phosphatase, γ-GTP: γ-glutamyl transpeptidase, BUN: blood urea nitrogen, LDH: Lactate dehydrogenase, CK: creatine kinase, CRP: C-reactive protein.

	Lab values	Reference values
Peripheral blood		
Hemoglobin	10.4 g/dL	13.5-17.0 g/dL
Hematocrit	31.2%	40-49.5%
White blood cells	9 800/μL	4 000 – 11 000/ μL
Platelets	429 000/μL	150 – 400/μL
AST	9 716 U/L	12-40 U/L
ALT	4620 U/L	7 - 40 U/L
ALP	89 U/L	46 - 116 U/L
γ-GTP	57 U/L	< 73 U/L
Total bilirrubin	0.31 mg/dL	0.2-1.1 mg/dL
BUN	52 mg/dL	19 – 49 mg/dL
LDH	5716 U/L	120 – 246 U/L
CK	258 U/L	46-171 U/L
Troponin I	0,057 ng/mL	< 0.045 ng/mL
CRP	137.70 mg/L	< 5.0 mg/L

The patient underwent an ECG in the emergency department, which revealed atrial fibrillation with a rapid ventricular response without signs suggestive of acute ischemia or electrical alternans.

Two weeks before, he was evaluated by cardiology, who identified a small-volume pericardial effusion and bilateral pleural effusion, both attributed to hypervolemia secondary to his ESRD.

**Figure 1:**
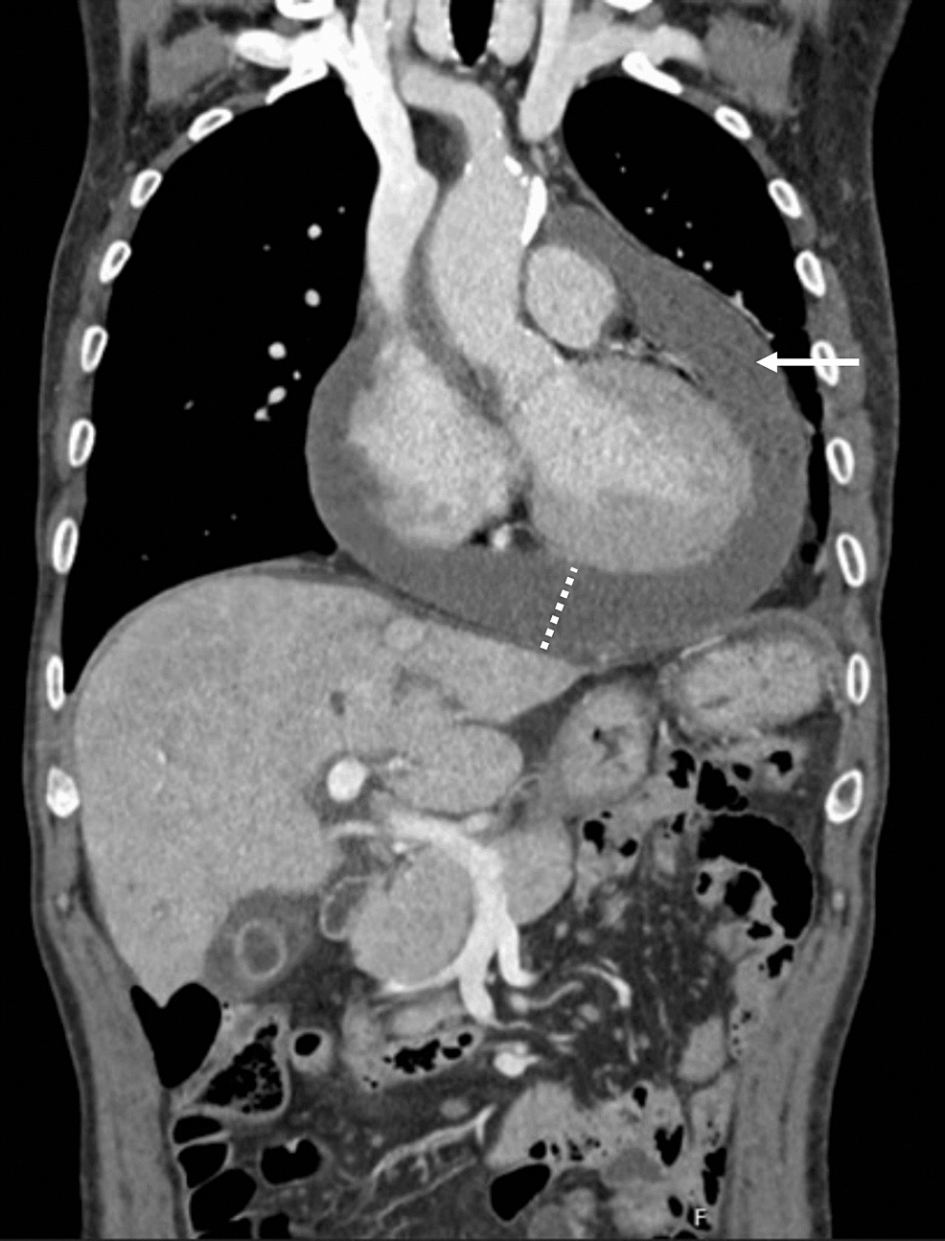
A coronal section of the thoracoabdominopelvic CT scan demonstrated pericardial enhancement compatible with active inflammation as well as a markedly large pericardial effusion.

Thoracoabdominopelvic CT scan (Figure 1) confirmed a pericardial effusion with a maximum thickness of 3.9 cm and small sub-centimetric hepatic cysts without other occupying lesions, as well as a small volume of ascites. Given this clinical picture, the patient was admitted to the intensive care unit (ICU) with the diagnosis of obstructive shock secondary to cardiac tamponade due to acute perimyocarditis of uncertain etiology complicated by ischaemic hepatitis.

During the stay in the ICU, the patient remained hypotensive, requiring noradrenaline support for six days (maximum dose of 0.6 mcg/kg/min). Pericardiocentesis was performed, with the drain remaining in place throughout the patient's ICU stay. On the sixth day, due to an improvement in blood pressure, the patient was transferred to the internal medicine ward. Subsequently, the results of the pericardial fluid laboratory study were obtained, revealing 4288 cells/μL (72% neutrophils), and cytological examination showed abundant neutrophils consistent with purulent pericarditis. Microbiological culture of the pericardial fluid isolated *Citrobacter freundii*. The patient was initiated on piperacillin-tazobactam treatment, which was later adjusted based on his antimicrobial susceptibility profile to cotrimoxazole.

After initiation of antibiotic therapy as well as a combination of acetylsalicylic acid and colchicine, it was possible to remove the pericardial drain (total drainage of 4445 mL over nine days, removed three days after the transfer to the internal medicine ward). The removal of the pericardial drain was prompted by the progressively decreasing volume of pericardial fluid drainage, which suggested a favorable clinical evolution. Further investigation for infectious causes of pericarditis and immune-mediated pericarditis is summarized in Table 2. The laboratory findings revealed slight complement C3 consumption and serological testing indicated past exposure to hepatitis A, Epstein-Barr virus, and parvovirus B19 and were not compatible with the recent infection.

**Table 2:** Pertinent laboratory values for acute pericarditis aetiological study. HAV: Hepatitis A virus; HCV: Hepatitis C virus, HIV: Human immunodeficiency virus, EBV: Epstein-Barr virus

	(i) Laboratory values	(ii) Reference values
Infectious serologies		
Hepatitis A		
Anti-HAV IgG	> 100 mIU/mL	Positive if > 20 mIU/mL
Anti-HAV IgM	0.17 S/CO	Negative if < 0.80 S/CO
Hepatitis B		
HBs antigen	Negative	-
HBs antibody	Positive	-
HBc antibody	Negative	-
Hepatitis C		
HCV antibody	Negative	-
HIV		
HIV I/II antibodies	Negative	-
Epstein-Barr Virus		
EBV early antigen IgG	Negative	-
EBV viral capside antigen IgG	Positive	-
EBV viral capside antigen IgM	Negative	-
EBV Nuclear antigen IgG	Positive	-
Leptospira		
Leptospira antibodies IgM	Negative	-
Parvovirus B19		
Parvovirus antibodies IgG	Positive	-
Parvovirus antibodies IgM	Negative	-
Coxiella burnetti		
Coxiella burnetti antibodies IgG/ IgM	Negative	-
Coxsackie virus		
Coxsackie virus antibodies IgG/ IgM	Negative	-
Immune-mediated disorder markers		
Rheumatoid factor	9 UI/mL	0-14 UI/mL
Antinuclear antibodies	Negative	-
Anti-smooth muscle antibodies	Negative	-
Anti-neutrophil cytoplasm antibodies	Negative	-
Anti-cyclic citrullinated peptide	Negative	-
Complement C4	13 mg/dL	10-40 mg/dL
Complement C3	72 mg/dL	90-180 mg/dL
Angiotensin-converting enzyme	54 U/L	20-70 U/L

In a follow-up evaluation, the patient remained asymptomatic for four weeks after continuing antibiotic therapy. A subsequent POCUS revealed only a thin layer of pericardial effusion, indicating a favorable resolution of the condition.

## Discussion

This case reports purulent pericarditis due to a *Citrobacter freundii* infection of the pericardial fluid. This diagnosis, severe in itself, is compounded by the fact that it occurred in an immunocompromised patient [3,4] who was also hypervolemic due to ESRD, which predisposed the superinfection of the pericardial fluid. The accumulation of pus in the pericardial space increased intrapericardial pressure to such an extent that it restricted cardiac chamber filling, reducing cardiac output (cardiac tamponade), thereby explaining the syncope due to cerebral hypoperfusion and the development of secondary ischemic hepatitis. Blood pressure and liver enzyme levels improved as the pericardial effusion was drained, but it was observed that the pericardial effusion recurred daily, with over 4 liters of pericardial fluid drained over nine days.

The favorable response to antibiotic treatment, coupled with the isolation of *Citrobacter freundii* in the pericardial fluid, makes the diagnosis of purulent pericarditis highly probable. Regarding the etiological agent, it is known to be a gram-negative bacterium of the *Enterobacteriaceae* family, which is associated with infections in patients with known comorbidities and immunocompromised patients [5], as in this particular case. Furthermore, there is a reported case of cardiac tamponade associated with purulent pericarditis caused by this microorganism, which occurred in a pediatric 10-year-old individual [5].

This case further emphasizes the critical importance that the treatment of acute pericarditis always presupposes identifying the underlying cause for a more appropriate approach to this pathology. As demonstrated in this case, pericardiocentesis is a vital diagnostic tool for determining the etiology, as documented in the literature through different clinical case reports [6-9].

## Conclusions

Purulent pericarditis remains a rare but serious complication, particularly in immunocompromised patients. Early recognition and aggressive management, including appropriate antibiotic therapy and drainage of pericardial effusion, are crucial for favorable outcomes. This case underscores the need for a high index of suspicion, thorough diagnostic evaluation, and a multidisciplinary management approach in similar clinical scenarios. Furthermore, it highlights the critical importance of identifying the etiology of pericarditis to guide targeted treatment strategies and prevent recurrence and complications. Addressing underlying immunosuppression and comorbidities is essential for comprehensive management and improved patient outcomes.

## References

[1] Lazarou E, Tsioufis P, Vlachopoulos C, Tsioufis C, Lazaros G (2022). Acute pericarditis: update. Curr Cardiol Rep.

[2] LeWinter MM (2014). Clinical practice. Acute pericarditis. N Engl J Med.

[3] Kaye A, Peters GA, Joseph JW, Wong ML (2019). Purulent bacterial pericarditis from Staphylococcus aureus. Clin Case Rep.

[4] Sagristà-Sauleda J, Barrabés JA, Permanyer-Miralda G, Soler-Soler J (1993). Purulent pericarditis: review of a 20-year experience in a general hospital. J Am Coll Cardiol.

[5] Warnow IE, Ayoola YA, Daniel A (2020). Citrobacter freundii: a cause of cardiac tamponade and empyema thoracis in a Nigerian child. J Cardiovasc Echogr.

[6] Aloqab F, Alsadah L, Shanei S (2023). A complex case of idiopathic purulent pericarditis in an immunocompetent adult. Cureus.

[7] Borkowski P, Borkowska N, Nazarenko N, Mangeshkar S, Akunor HS (2024). Hemopericardium: a comprehensive clinical review of etiology and diagnosis. Cureus.

[8] Mascarenhas L, Agakishiev D, Freeman M, Hubers S (2024). Purulent pericarditis caused by methicillin-sensitive Staphylococcus aureus bacteriuria. BMC Cardiovasc Disord.

[9] Narang VK, Bowen J, Masarweh O (2022). Acute pericarditis leading to a diagnosis of SLE: a case series of 3 patients. J Investig Med High Impact Case Rep.

